# Beneficial and Detrimental Effects of Reactive Oxygen Species on Lifespan: A Comprehensive Review of Comparative and Experimental Studies

**DOI:** 10.3389/fcell.2021.628157

**Published:** 2021-02-11

**Authors:** Hazel J. Shields, Annika Traa, Jeremy M. Van Raamsdonk

**Affiliations:** ^1^Department of Neurology and Neurosurgery, McGill University, Montreal, QC, Canada; ^2^Metabolic Disorders and Complications Program, Research Institute of the McGill University Health Centre, Montreal, QC, Canada; ^3^Brain Repair and Integrative Neuroscience Program, Research Institute of the McGill University Health Centre, Montreal, QC, Canada; ^4^Division of Experimental Medicine, Department of Medicine, McGill University, Montreal, QC, Canada; ^5^Department of Genetics, Harvard Medical School, Boston, MA, United States

**Keywords:** aging, reactive oxygen species, free radical theory of aging, genetics, lifespan, antioxidants, model organisms

## Abstract

Aging is the greatest risk factor for a multitude of diseases including cardiovascular disease, neurodegeneration and cancer. Despite decades of research dedicated to understanding aging, the mechanisms underlying the aging process remain incompletely understood. The widely-accepted free radical theory of aging (FRTA) proposes that the accumulation of oxidative damage caused by reactive oxygen species (ROS) is one of the primary causes of aging. To define the relationship between ROS and aging, there have been two main approaches: comparative studies that measure outcomes related to ROS across species with different lifespans, and experimental studies that modulate ROS levels within a single species using either a genetic or pharmacologic approach. Comparative studies have shown that levels of ROS and oxidative damage are inversely correlated with lifespan. While these studies in general support the FRTA, this type of experiment can only demonstrate correlation, not causation. Experimental studies involving the manipulation of ROS levels in model organisms have generally shown that interventions that increase ROS tend to decrease lifespan, while interventions that decrease ROS tend to increase lifespan. However, there are also multiple examples in which the opposite is observed: increasing ROS levels results in extended longevity, and decreasing ROS levels results in shortened lifespan. While these studies contradict the predictions of the FRTA, these experiments have been performed in a very limited number of species, all of which have a relatively short lifespan. Overall, the data suggest that the relationship between ROS and lifespan is complex, and that ROS can have both beneficial or detrimental effects on longevity depending on the species and conditions. Accordingly, the relationship between ROS and aging is difficult to generalize across the tree of life.

## Introduction

Aging can be described as the gradual loss of fitness due to detrimental changes occurring at the cell and molecular level over time. It is characterized by dysregulation of cellular processes, accumulation of damaged materials and toxins, altered gene expression, and poor immune and stress responses (Partridge and Gems, 2002; Kennedy et al., 2014; DiLoreto and Murphy, 2015). While the changes that occur during the aging process have been well described, the mechanisms underlying aging remain poorly understood, and have thus been the subject of much research. One of the most widely accepted theories of aging, called the free radical theory of aging (FRTA), proposes that oxidative damage caused by reactive oxygen species (ROS) is the primary cause of aging. While there have been many studies examining the relationship between ROS and aging, this is still an area of much debate. In this work, we review comparative studies and studies involving experimental modulation that have investigated the role of ROS in aging across species. Overall, these studies demonstrate that the relationship between ROS and aging is complex, in that ROS can both increase or decrease lifespan depending on the experimental conditions.

### Reactive Oxygen Species

Reactive oxygen species are highly reactive, oxygen containing molecules that are the result of an incomplete reduction of molecular oxygen in the cell (Santos et al., 2018). ROS can be free radicals, or molecules that have the capacity to generate free radicals. Free radicals consist of atoms or molecules with an unpaired electron in their outer shell causing them to be unstable and highly reactive, or in other words, prone to “stealing” electrons from other molecules (Pham-Huy et al., 2008). Free radicals are highly reactive and are therefore generally short-lived and often unable to leave the subcellular location where they are generated without first becoming reduced. Examples of ROS that are free radicals include the superoxide (${\text{O}}_{2}^{•-}$), hydroxyl (HO^•^), peroxyl (${\text{RO}}_{2}^{•-}$), hydroperoxyl (${\text{HO}}_{2}^{•}$), and alkoxyl radicals (RO^•^) (Winterbourn, 2008; Phaniendra et al., 2015). ROS that are not free radicals do not have unpaired electrons, and are often less reactive, thus allowing them to leave the subcellular location where they are generated as well as pass through membranes (Winterbourn, 2008). Examples of ROS that are not free radicals include hydrogen peroxide (H_2_O_2_), the hydroxide ion (OH^–^) and organic peroxides (ROOH) (Phaniendra et al., 2015).

**FIGURE 1:**
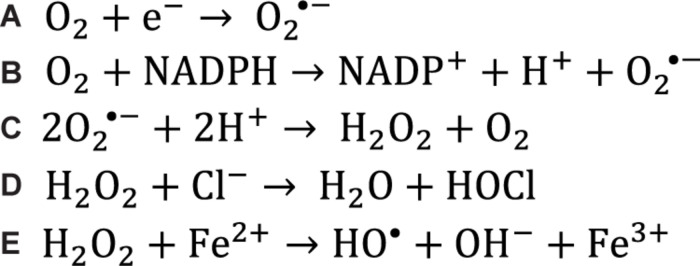
Chemical reactions that generate reactive oxygen species. **(A)** Superoxide is generated in the mitochondria when electrons leak out of the electron transport chain and reduce singlet oxygen. **(B)** Superoxide can also be generated in the cell when enzymes catalyze the transfer of an electron from NADPH to singlet oxygen, often during metabolism reactions. **(C)** Two superoxide molecules can be converted to hydrogen peroxide and oxygen by the superoxide dismutase enzymes. **(D)** Myeloperoxidase catalyzes the conversion of hydrogen peroxide and a chloride anion to hypochlorous acid which acts as a potent oxidizer in the respiratory burst. **(E)** When hydrogen peroxide encounters free ferrous iron within the cell, the Fenton reaction occurs, producing a hydroxyl radical.

Despite being less reactive, ROS that are not free radicals are still incompletely reduced and thus can undergo redox reactions to produce free radicals as a result. For example, if hydrogen peroxide encounters a reduced transition metal ion such as ferrous iron (Fe^2+^) or cuprous copper (Cu^+^), the Fenton reaction will occur (Figure 1), resulting in the production of the hydroxyl radical which acts as a potent oxidant (Sutton and Winterbourn, 1989). The ability for ROS that are not free radicals, such as hydrogen peroxide, to move within the cell becomes important when considering the redox state for cell signaling in various subcellular locations as well as antioxidant distribution within the cell (Travasso et al., 2017; Di Marzo et al., 2018).

In addition to ROS, organisms also generate reactive nitrogen species (RNS). Nitric oxide (NO) is produced from L-arginine by the enzyme nitric oxide synthase of which there are three forms: endothelial (eNOS), inducible (iNOS) and neuronal (nNOS). Nitric oxide can then react with superoxide to produce peroxynitrite (ONOO^–^), which can directly damage cellular components, or further react to generate other types of RNS. As with ROS, there are both free radical forms of RNS [nitric oxide, nitric dioxide (NO_2_⋅), nitrate radical (NO_3_⋅)] and non-radical forms [peroxynitrite, nitrous acid (HNO_2_), nitrite (NO_2_−), nitrosyl cation (NO+), nitroxyl anion (NO−), peroxynitrous acid (ONOOH), dinitrogen trioxide (N_2_O_3_)]. Also similar to ROS, RNS can cause cellular damage called nitrosative damage, but also have functional roles in cellular signaling and pathogen defense. Little is known about the effects of RNS on longevity.

### Oxidative Damage as a Biomarker of Aging

**FIGURE 2:**
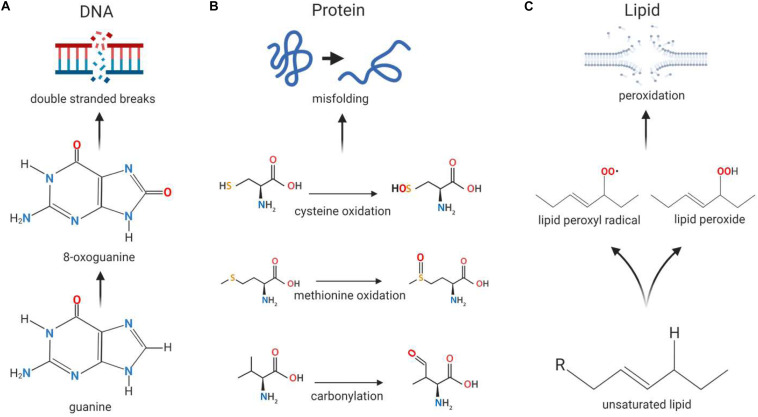
Macromolecular damage caused by reactive oxygen species. Reactive oxygen species (ROS) can cause damage to the basic building blocks of the cell including DNA, protein and lipids. **(A)** DNA damage can occur in the form of double stranded breaks as a result of ROS-induced conversion of guanine to 8-oxoguanine. As 8-oxoguanine can be mis-paired with adenine, transversion mutations can occur, resulting in double stranded breaks after replication. **(B)** Proteins can become misfolded when exposed to ROS due to oxidation of amino acids cysteine and methionine, as well as carbonylation of the peptide backbone, resulting in changes to the molecular interactions that normally occur within the peptide. **(C)** Exposure to ROS can induce membrane damage when lipid peroxidation occurs as a result of the formation of lipid peroxides and lipid peroxide radicals, the latter of which can cause further oxidative damage to other lipids.

The tendency for free radicals to steal electrons in order to stabilize themselves can be problematic in cells, where they may cause oxidative damage to macromolecules such as DNA, proteins and lipids (Pham-Huy et al., 2008). When DNA is exposed to ROS, guanine is modified to 8-oxoguanine (Figure 2A), allowing it to pair with cytosine and adenine. This mutation can occur in both nuclear and mitochondrial DNA and can give rise to double stranded breaks (DSBs) in the DNA, leading to genomic instability (Kregel and Zhang, 2007). Proteins can be damaged when amino acid side chains and backbones, especially in thiol-containing cysteine and methionine residues, are oxidized by ROS (Figure 2B); this can result in structural changes to the protein that may lead to loss of function (Sohal, 2002) or be used for ROS-mediated signaling. Additionally, the exposure of lipids to ROS results in lipid peroxidation (Figure 2C), which gives rise to cell membrane damage and generates reactive by-products which can further damage the cell (Mylonas and Kouretas, 1999).

All of these types of oxidative damage have been shown to increase with advancing age including DNA damage (Massudi et al., 2012; Moskalev et al., 2013), protein carbonylation (Moskovitz and Oien, 2010; Jha and Rizvi, 2011), lipid peroxidation (Massudi et al., 2012) and damage to mitochondrial DNA (Park and Larsson, 2011). As a result, these outcome measures have been proposed as biomarkers of aging [reviewed elsewhere e.g. (Syslova et al., 2014; Liguori et al., 2018)].

### The Free Radical Theory of Aging

The FRTA proposes that aging is caused by the accumulation of molecular damage caused by ROS that are generated by normal metabolism (Harman, 1956). The logic of the FRTA stems from the idea that ROS have the capacity to be highly damaging to cell components, as well as the idea that the mechanism of aging is likely to be an intrinsic process given that aging always occurs, regardless of differences in environment. The FRTA developed into the mitochondrial FRTA (MFRTA) which became a popular way to explain why the rate of aging and the maximum lifespan varies so significantly among species (Barja, 2013). The MFRTA specifies that the ROS that cause aging are produced by the mitochondria, and that the lifespan of an organism thereby depends on their rate of oxygen consumption by the mitochondrial respiratory chain, which is believed to be the main process that produces ROS. The theory fit with the idea that the mechanism of aging is intrinsic, given the constant endogenous production of ROS as a byproduct of a process essential to all known aerobic organisms.

Initially proposed by Harman (1956) and revised in Harman (1972), the FRTA has since been supported by observations on the association between ROS, oxidative damage and longevity. These observations include: (1) higher levels of ROS generation with increasing age (Sawada and Carlson, 1987; Sohal and Sohal, 1991; Sohal and Dubey, 1994; Bejma and Ji, 1999; Driver et al., 2000; Capel et al., 2005); (2) higher levels of oxidative damage with increasing age (Oliver et al., 1987; Fraga et al., 1990; Hamilton et al., 2001; Bokov et al., 2004; Navarro and Boveris, 2004; Muller et al., 2007; Halliwell, 2009); (3) gradual increase in mitochondrial dysfunction with age (Rockstein and Brandt, 1963; Trounce et al., 1989; Cooper et al., 1992; Genova et al., 2004; Green et al., 2011; Gouspillou et al., 2014; Sun et al., 2016); (4) an increase in ROS generation upon inhibition of components of the electron transport chain (Turrens and Boveris, 1980; Forman and Azzi, 1997; Kushnareva et al., 2002; Chen et al., 2003; Li et al., 2003; Fato et al., 2009); and (5) high levels of oxidative stress in several age-related diseases (Stohs, 1995; Berliner and Heinecke, 1996; Forsberg et al., 2001; Oberley, 2002; Lassègue and Griendling, 2004; Cutler, 2005; Chung et al., 2006; Pham-Huy et al., 2008).

In addition to support for the FRTA, observations refuting the theory have also accumulated (Yang et al., 2007; Van Raamsdonk and Hekimi, 2009; Yang and Hekimi, 2010; Van Raamsdonk and Hekimi, 2012; Wang et al., 2018), with recent findings ultimately leading to the formation of new models for the relationship between ROS, redox signaling, oxidative damage and lifespan (Lapointe and Hekimi, 2010; Van Raamsdonk and Hekimi, 2010; Hekimi et al., 2011). Newer models for the role of ROS in the cell suggest that a mild elevation in ROS can be beneficial to an organism, perhaps through the activation of cellular stress response signaling pathways, while very low and very high levels can be detrimental to organisms (Hekimi et al., 2011; Sena and Chandel, 2012; Van Raamsdonk, 2015). These models incorporate the importance of the maintenance of adequate ROS levels for redox signaling components (ex. thiol-containing proteins) which may rely on ROS-mediated oxidation in order to activate desired cell survival pathways (Sundaresan et al., 1995; McCord, 2000; Woo et al., 2000; Dröge, 2002; Theopold, 2009; Flohé, 2010; Ray et al., 2012).

### Sources of Reactive Oxygen Species in the Cell

**FIGURE 3:**
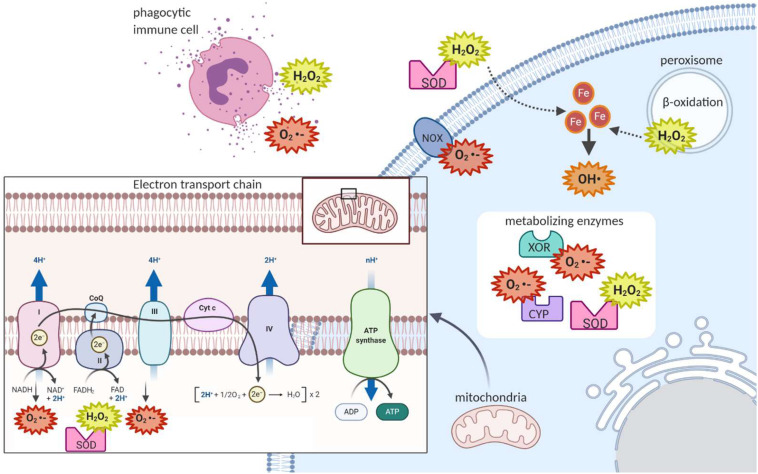
Sites of reactive oxygen species generation in the cell. As electrons are being passed from Complex I or Complex II to Complex III via ubiquinone in the mitochondrial electron transport chain, some of these electrons can escape and react with oxygen to form superoxide. The enzymes superoxide dismutase can convert the superoxide to hydrogen peroxide, which can then exit the mitochondria. In the cytoplasm, metabolism reactions such as those of the cytochrome p family of enzymes (CYP) produce ROS. In the peroxisome, fatty acid beta-oxidation produces hydrogen peroxide. At the plasma membrane, NADPH Oxidase produces superoxide. Extracellularly, ROS can be released in processes such as the respiratory burst, where phagocytic immune cells release ROS to attack pathogens. Extracellular superoxide dismutase can then convert extracellular superoxide to hydrogen peroxide. Hydrogen peroxide, which can cross membranes, can be converted to the potent hydroxyl radical when in contact with cellular ferrous iron.

Mitochondrial respiration is a major source of ROS within the cell. As electrons are transferred between the complexes of the electron transport chain, some of these electrons can “leak” out to react directly with oxygen to form superoxide (Figures 1A and 3) (Kushnareva et al., 2002; Chen et al., 2003; Balaban et al., 2005). The majority of electron leakage is thought to occur as electrons are passed from Complex I or Complex II to Complex III via ubiquinone (Van Raamsdonk and Hekimi, 2010).

There are also important non-mitochondrial sources of ROS in the cell. For example, immune cells attack pathogens in the body by releasing ROS extracellularly or into the phagolysosome, a strategy termed the respiratory burst (Figure 3) (Beckman and Ames, 1998). The ROS used in this attack are generated by the membrane-bound phagocyte NADPH oxidase as well as the granule-localized myeloperoxidase (Nguyen et al., 2017). NADPH oxidase uses the electrons donated from NADPH to convert molecular oxygen to a superoxide anion (Figure 1B), while myeloperoxidase (MPO) converts hydrogen peroxide to hypochlorous acid (Figure 1D) (Nguyen et al., 2017). Importantly, NADPH oxidase homologs (NOX enzymes; Figure 3) expressed in other cell types also play important roles in the generation of ROS for signal transduction in pathways for growth, survival and apoptosis (Lambeth, 2020).

In fact, there are many ROS-generating enzymes that play essential roles in various physiological systems (Figure 3). Some examples include: the cytochrome P450 (CYP) family of enzymes, which produce ROS in the process of the detoxification and excretion of xenobiotics (McDonnell and Dang, 2013); xanthine oxidoreductase (XOR), which produces superoxide anions during the break down of purines to uric acid (Battelli et al., 2016); superoxide dismutase (SOD), which converts superoxide to hydrogen peroxide and oxygen (Figure 1C) (McCord and Fridovich, 1969); and monoamine oxidase, which breaks down the neurotransmitter dopamine after signaling occurs and produces hydrogen peroxide in neurons in the process (Shih et al., 1999).

Finally, an additional source of ROS comes from β-oxidation of fatty acids in the peroxisome, producing hydrogen peroxide which can then cross the plasma membrane of the peroxisome, to reach the cytoplasm (Figure 3) (Beckman and Ames, 1998). Along with NADPH oxidase-generated ROS, peroxisome-generated ROS are thought to contribute to regulation of the activity of NF-kB and mTORC1 which both play important roles in cell survival in response to stressors (Lismont et al., 2015).

### Detoxification of Reactive Oxygen Species

Reactive oxygen species levels in the cell can be seen as a balance between levels of ROS generation and levels of ROS detoxification by antioxidants. Maintaining homeostatic levels of ROS as a result of this balance is important both for preventing oxidative damage as well as maintaining an appropriate redox environment for normal signaling pathways within the cell (Forman et al., 2014). Thus, just as ROS detoxification is required to avoid increases in ROS levels, antioxidant activity must be regulated to avoid over-detoxification and low levels of ROS. In response to an imbalanced redox environment, gene expression of antioxidant enzymes is triggered (Ma, 2013). Antioxidants scavenge ROS through various reactions which facilitate the reduction of ROS to less reactive and more stable forms (Matés et al., 1999). Enzymatic antioxidants are localized to various subcellular locations in order to be in proximity to sites of ROS generation. Non-enzymatic antioxidants can be endogenous or supplied by dietary nutrients (Matés et al., 1999).

#### Enzymatic Antioxidants

##### Superoxide dismutase

**FIGURE 4:**
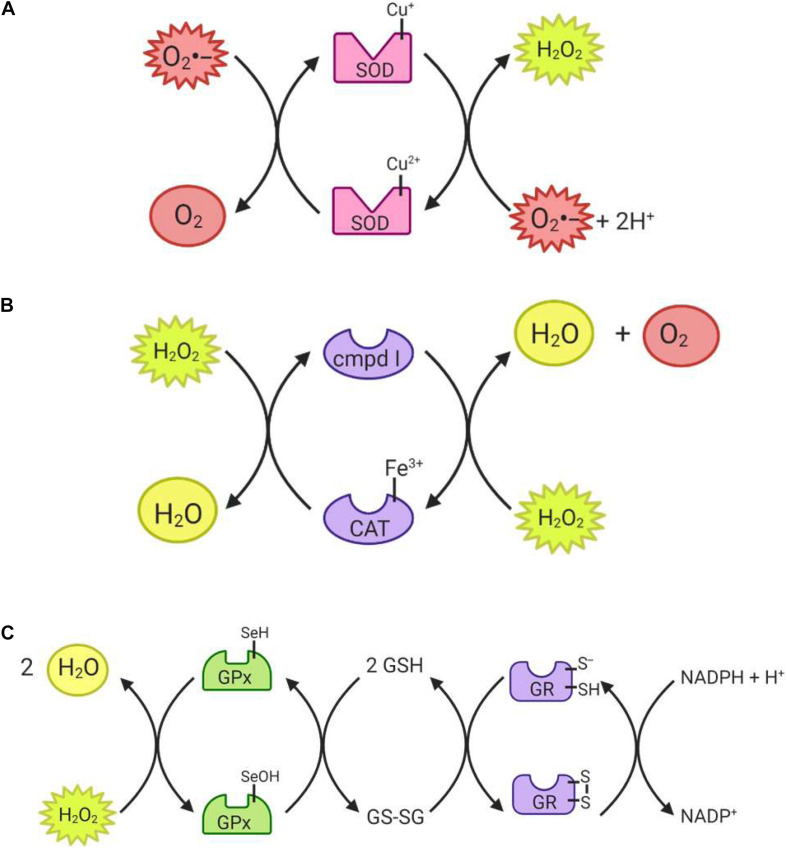
Reactions catalyzed by antioxidant enzymes I. **(A)** Superoxide dismutase (SOD) uses either a reduced copper or manganese active site to reduce two superoxide radicals, producing hydrogen peroxide in the process. **(B)** Catalase (CAT) can convert hydrogen peroxide to water, and is itself converted to compound I in the process. In order to return to its active state, the enzyme must reduce another hydrogen peroxide molecule to water and oxygen. **(C)** Glutathione peroxidase (GPx) can reduce both hydrogen peroxide and organic peroxides using its selenocysteine active site. The enzyme then returns to its active state through reduction by glutathione (GSH). Glutathione reductase (GR) can then reduce glutathione disulfide (GSSG) back to GSH molecules.

**FIGURE 5:**
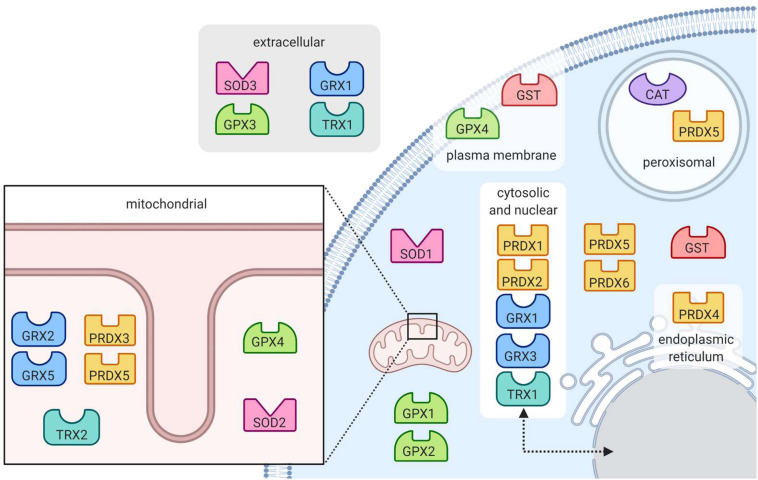
Location of antioxidant enzymes within the cell. Superoxide dismutase enzymes (SOD) are found in the mitochondria, cytoplasm and extracellular space. Catalase (CAT) works to eradicate hydrogen peroxide in the peroxisome. Peroxide-reducing peroxiredoxins (PRX) are present throughout the cell, notably in peroxisomes. Also reducing peroxides, glutathione peroxidases (GPX) are present in the mitochondria and cytoplasm, at the plasma membrane and in the extracellular space. Protein-protecting glutaredoxins (GRX) and thioredoxins (TRX) are present in many subcellular locations including in the mitochondria, cytoplasm, nucleus, and extracellular space. Glutathione *s*-transferases (GST) are located in the cytoplasm and at the plasma membrane.

The superoxide dismutase (SOD) enzyme converts the highly reactive superoxide anion to a less reactive hydrogen peroxide. The enzyme catalyzes the transfer of electrons through two redox reactions using transition metals in its active site (Tainer et al., 1983). In the first reaction, superoxide is oxidized to molecular oxygen, leaving the transition metal in the active site reduced; in the second reaction, superoxide is reduced to hydrogen peroxide and the transition metal in the active site returns to its oxidized form (Figure 4A) (McCord and Fridovich, 1969). Humans have three different forms of SOD (Figure 5): SOD1 is located in the cytosol and its active site is Cu/Zn; SOD2 is located in the mitochondria and its active site is Mn; and SOD3 is located extracellularly with a Cu/Zn active site (Fukai and Ushio-Fukai, 2011).

##### Catalase

Catalase (CAT) catalyzes the conversion of hydrogen peroxide to water and molecular oxygen (Figure 4B) and thus plays an important role in dealing with hydrogen peroxide-producing reactions, including that of the SODs (Ighodaro and Akinloye, 2019). Its active site is composed of heme groups which facilitate the transfer of electrons. Catalase is primarily located in the peroxisomes (Figure 5), where hydrogen peroxide production can be high due to β-oxidation (Birben et al., 2012; Ighodaro and Akinloye, 2019).

##### Glutathione peroxidase

Glutathione peroxidase (GPx) functions in conjunction with catalase to reduce hydrogen peroxide levels in the cell. GPx is located in the cytoplasm and mitochondria, while catalase is mainly in peroxisomes (Baud et al., 2004; Ighodaro and Akinloye, 2019). While catalase mainly detoxifies hydrogen peroxide, GPx is also able to detoxify various peroxides, notably lipid peroxides (Ighodaro and Akinloye, 2019). In the reaction to reduce hydrogen peroxide (Figure 4C), the selenocysteine active site of GPx becomes oxidized. In order to return the active site to its functional state, two glutathione (GSH) molecules are needed (Flohé, 1985), resulting in the production of glutathione disulfide (GS-SG). Given that GSH is essential to maintain the activity of GPx, GS-SG must be reduced back to two molecules of GSH; this is catalyzed by glutathione reductase (GR), and uses electrons donated by NADPH (Flohé, 1985; Ursini et al., 1985). The GPx enzymes act in multiple subcellular compartments (Figure 5): GPX1 and GPX2 deal with hydrogen peroxide and organic peroxides in the cytosol, GPX3 acts as an extracellular antioxidant and GPX4, which can metabolize phospholipid hydroperoxides, is located in cell membranes and in mitochondria (Arthur, 2000).

##### Glutaredoxin and thioredoxin

**FIGURE 6:**
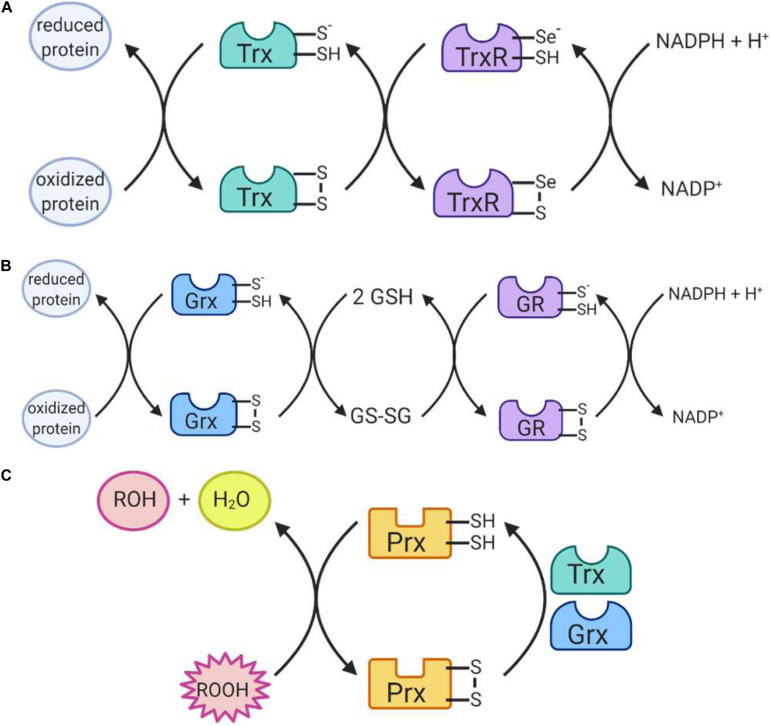
Reactions catalyzed by antioxidant enzymes II. **(A)** Thioredoxin (Trx) uses its thiol containing active site to return proteins to a reduced state. Trx itself is then reduced by the selenium-containing active site of thioredoxin reductase (TrxR), which is in turn reduced by NADPH. **(B)** Glutaredoxin (Grx) also uses its thiol-containing active site to reduce proteins. The enzyme is then reduced by 2 glutathione (GSH) molecules, producing glutathione disulfide (GSSG). GSSG is reduced by the thiol-containing active site of glutathione reductase (GR) which is in turn reduced by NADPH. **(C)** Peroxiredoxins (Prx) work to reduce hydrogen peroxide and organic peroxides using its thiol-containing active site. The enzyme is itself then reduced by Trx or Grx to return to its active state.

The glutaredoxin (Grx) and thioredoxin (Trx) family of antioxidant enzymes act to protect thiol-containing proteins by repairing damage caused by exposure to oxidants. This system catalyzes the transfer of electrons to the disulfide bonds of target proteins, in order to convert the target protein back to its reduced form (Lee et al., 2013). This is done through a series of reactions (Figures 6A,B), which begin with the reduction of the target residue, and oxidation of the Grx or Trx active site, which consist of cysteine residues (Hanschmann et al., 2013). Each enzyme then needs to be reduced back to its active form: Grx is reduced by two GSH molecules, which are in turn oxidized to glutathione disulfide while thioredoxin is reduced by an enzyme called thioredoxin reductase (TrxR) (Hanschmann et al., 2013). TrxR then harnesses electrons donated by NADPH molecules to reduce its own thiol-containing active site back to its active state while glutathione disulfide is reduced back to two GSH molecules by the GR, which also harnesses electrons from NADPH (Lee et al., 2013). Importantly, both enzymes are recognized as playing important roles in redox signaling pathways within the cell, especially given their abundance throughout the cell (Figure 5). In mammalian cells, for example, TRX1 and GRX1 are both located in the cytosol, nucleus and can be secreted, TRX2 and GRX2 are located in the mitochondria, GRX3 is also located in the cytosol and nucleus and GRX5 is in the mitochondria (Hanschmann et al., 2013).

##### Peroxiredoxin

The family of peroxiredoxin (Prx) enzymes are cysteine-containing enzymes that reduce peroxides, including organic peroxides (Figure 6C) (Perkins et al., 2015). Several Prx enzymes are located throughout the cell (Figure 5): in mammals, PRDX1 and PRDX2 are located in the cytosol and nucleus, PRDX3 in the mitochondria, PRDX4 in the endoplasmic reticulum, PRDX5 in peroxisomes, mitochondria and cytosol, and PRDX6 in the cytosol (Hanschmann et al., 2013; Rhee, 2016). Once the cysteine-containing active site reduces the target peroxide, Prx enzymes are returned to their active state when reduced by either Trx or GSH (Perkins et al., 2015).

##### Glutathione S-transferase

Glutathione *S*-transferases (GSTs) have been linked to various stress responses and are thought to help GPx in attenuating lipid peroxidation by reducing fatty acid hydroperoxides (Sharma et al., 2004). The enzyme does so by binding GSH and catalyzing the transfer of electrons to the target lipid, its active site consisting of a serine residue (Sharma et al., 2004; Dixon et al., 2011). In addition to preventing lipid peroxidation, GSTs may also help detoxify the end products of lipid peroxidation, which can be very harmful to the cell. There are many GST isozymes, most of which are in the cytosol, microsomes and plasma membrane (Figure 5) (Sharma et al., 2004).

#### Non-enzymatic Antioxidants

##### Glutathione

Appropriate glutathione (GSH) levels are essential for the activity of the GPx and Grx systems, where GSH acts to return each enzyme back to its active state (Flohé, 1985; Birben et al., 2012). In addition, GSH itself can act as an antioxidant using the sulfhydryl group to donate electrons in order to reduce and detoxify ROS (Birben et al., 2012). Levels of active GSH can be restored either by converting oxidized GSH back to its reduced state by GR and NADPH, or by dietary supplementation (Wu et al., 2004). GSH can be added to oxidized cysteine residues in proteins through the process of *S*-glutathionylation. Because this process is reversible and can modify protein function, glutathionylation can be used in intracellular signaling (Xiong et al., 2011). Interestingly, the levels of glutathione can be increased by the endogenously-produced signaling molecule hydrogen sulfide (H_2_S) (Kimura et al., 2010), which has also been shown to increase lifespan (Miller and Roth, 2007).

##### N-acetyl cysteine

Not only is *N*-acetyl cysteine (NAC) a precursor molecule in the formation of GSH, it also has its own thiol group, allowing it to participate in redox reactions and donate its electrons for the detoxification of ROS and the protection of sulfhydryl-containing proteins from oxidative damage (Aldini et al., 2018). NAC levels are maintained by the consumption of cysteine in high protein foods (Salamon et al., 2019).

##### Vitamin C and vitamin E

Vitamin C (ascorbic acid) is a water soluble antioxidant that is present both intracellularly and extracellularly. It acts by scavenging oxygen free radicals in aqueous environments, and by providing a line of defense against oxidation of cholesterols (Frei, 1994). Vitamin E (alpha-, beta-, gamma-, delta-tocopherols, and alpha-, beta-, gamma-, delta-tocotrienols) is lipid soluble and present in cell membrane and lipoproteins, working to inhibit lipid peroxidation as a result of oxidative stress (Frei, 1994). Vitamin C can help convert Vitamin E back to it reduced form (Birben et al., 2012).

#### Repair of Oxidative Damage

While antioxidant systems work to balance ROS levels and prevent oxidative damage, the cell also employs various repair systems to reverse damaging oxidation of cell components. As described above, glutaredoxins and thioredoxins play an important role in the reduction of thiol-containing proteins. Additional enzymes work alongside thioredoxins such as thioredoxin-related protein of 14 kDa and methionine sulfide reductase to reduce cysteine and methionine residues, respectively, both of which are amino acids that are most susceptible to oxidation by ROS (Moskovitz et al., 2001; Pader et al., 2014). DNA damage repair mechanisms can be trigged by redox sensors which activate the expression of enzymes mediating base excision repair (BER), nucleotide excision repair (NER) and mismatch repair. The common DNA oxidation product, 8-oxoguanine, is repaired by 8-oxoguanine glycosylase (OGG1) via base excision (Van Houten et al., 2018).

In addition to mechanisms which work to reverse oxidative damage, quality control systems which prevent the over-accumulation of oxidatively damaged molecules and organelles have been described. Irreversibly oxidized proteins are targeted by unfolded protein response machinery and can be degraded via the proteasome in the cytosol or by proteases in the mitochondria (Friguet, 2006; Lionaki and Tavernarakis, 2013). Furthermore, oxidative stress may trigger autophagy of peroxisomes (pexophagy) and mitochondria (mitophagy) allowing for the removal of dysfunctional and ROS-generating organelles, followed by the synthesis of new undamaged organelles (Lemasters, 2005).

### Measuring Reactive Oxygen Species

While the short lifespan and high reactivity of ROS make them difficult to measure accurately, especially *in vivo*, a number of methods to measure ROS directly have been developed [for more comprehensive reviews of approaches to measure ROS see (Dikalov and Harrison, 2014; Kaludercic et al., 2014; Pavelescu, 2015; Griendling et al., 2016)]. Many of these approaches rely on molecules that emit fluorescence after they have been oxidized by ROS. Among the most utilized of these molecules are 2′,7′-dichlorodihydrofluorescein diacetate (DCF), dihydroethidium (DHE), and MitoSOX, which are believed to measure overall cellular ROS (Wang and Roper, 2014), cellular superoxide levels, and mitochondrial superoxide levels, respectively (Wang and Zou, 2018). Similarly, Amplex Red has been used extensively to measure hydrogen peroxide levels. However, there is much disagreement about the sensitivity and specificity of these approaches especially given the fact that these molecules can also participate in non-specific redox reactions, producing a different but similarly fluorescent adduct that can be hard to distinguish from the fluorescent products derived from reactions with ROS (Wang and Zou, 2018). Furthermore, these molecules are membrane permeable and can leak out of the cell, posing a problem when measuring cellular ROS in tissues (Wang and Roper, 2014).

Given the difficulties in directly measuring ROS levels, investigators often instead quantify ROS indirectly, either by assessing oxidative damage to cell components, or measuring survival under conditions of oxidative stress (Katerji et al., 2019). Levels of oxidative damage have most commonly been assessed by measuring the levels of 8-oxoguanine residues in DNA, carbonylation of protein, or levels of malondialdehyde (MDA), an end product of lipid peroxidation (Figure 2) (Esterbauer and Cheeseman, 1990; Ock et al., 2012; Mesquita et al., 2014). Increased levels of oxidative damage are generally interpreted to indicate increased levels of ROS; however, this outcome could also result from decreased levels of antioxidant or repair enzymes. To quantify resistance to oxidative stress, animals can be treated with ROS-generating compounds such as paraquat, juglone, and menadione (Fukushima et al., 2002; Criddle et al., 2006; Loor et al., 2010; Anaissi-Afonso et al., 2018; Ahmad and Suzuki, 2019). Decreased resistance to oxidative stress may be an indication of elevated baseline levels of ROS; however, increased levels of ROS can also cause upregulation of antioxidant enzymes, and thus resistance to oxidative stress must be interpreted with great care.

More recently, genetically-encoded redox sensors have been developed to measure ROS *in vivo* without the spatial and temporal restrictions encountered with other ROS detection methods (Chiu et al., 2014; Kaludercic et al., 2014; Erard et al., 2018). These sensors involve the expression of a redox-sensitive protein that emits fluorescence after it encounters ROS. These sensors are minimally invasive, can be stably expressed in specific tissues, can be targeted to specific sub-cellular locations, and can be used for real-time ROS detection *in vivo* (Erard et al., 2018). Given that an increasing body of work shows that the specific conditions (e.g., timing, sub-cellular location, and levels) determine whether ROS have beneficial or detrimental effects, genetically-encoded redox sensors are expected to provide a highly useful tool for future studies on the role of ROS in aging and disease (Chiu et al., 2014; Erard et al., 2018).

## Role of ROS in Determining Longevity: Evidence by Association

In order to study the relationship between ROS and aging, many groups have utilized a comparative biology approach in which the levels of ROS, or ROS-related outcomes, are measured across species with differing lifespans. In examining the relationship between lifespan and either ROS levels, antioxidant levels or oxidative damage, most studies have demonstrated a negative correlation. While this comparative data is generally supportive of the FRTA, exceptions and contradictions have also been observed.

### ROS Production Is Negatively Correlated With Longevity

Across species with varying lifespan, data collected from comparative studies shows a negative correlation between the rate of mtROS production and lifespan (Table 1). Comparing the superoxide and hydrogen peroxide generation rates across seven mammalian species revealed an inverse relationship between ROS production rates and lifespan (Sohal et al., 1989; Ku et al., 1993). Furthermore, a similar study comparing five different species of dipteran flies supported these findings, showing that longer lifespan correlates with relatively low levels of ROS generation (Sohal et al., 1995). In these studies, measurement of superoxide production was carried out in submitochondrial particles by looking at the reduction of cytochrome *c* due to its interaction with superoxide, while hydrogen peroxide generation was measured fluorometrically by monitoring the oxidation of *p*-hydroxyphenylacetate (PHPA) coupled to the enzymatic reduction of H_2_O_2_ by horseradish peroxidase (Sohal et al., 1989, 1995; Ku et al., 1993).

**TABLE 1:** Levels of reactive oxygen species are negatively correlated with longevity in comparative studies.

ROS being measured	Experimental animals	Range of lifespans	Finding	References
H_2_O_2_, O_2_^–^	Flies	29.5–65.5 days	Negative correlation	Sohal et al., 1995
H_2_O_2_, O_2_^–^	Pigeons and rats	4.5–35 years	Negative correlation	Ku and Sohal, 1993
H_2_O_2_, O_2_^–^	Mammals	3.5–30 years	Negative correlation	Ku et al., 1993
H_2_O_2_	Mammals and birds	3.5–37.5 years	Negative correlation	Lambert et al., 2007
O_2_^–^	Mammals and flies	3 months–30 years	Negative correlation	Sohal et al., 1989

Despite seemingly supportive evidence from these studies, it was found that metabolic rate also correlated with lifespan (Sohal et al., 1989; Ku et al., 1993). In order to isolate which factor is responsible for the observed results, the levels of mtROS production were compared between species with the same metabolic rate but different lifespans. While pigeons and rats are similar in size and have the same metabolic rate, pigeons live 35 years whereas rats only live 4 years. When superoxide and hydrogen peroxide generation rates where studied in the brain, heart, and kidney of these two animals, it was found that the pigeon had a significantly lower ROS generation rate (Ku and Sohal, 1993), again supporting a negative relationship between ROS production and lifespan.

While many studies have observed the same inverse relationship between lifespan and the rate of ROS generation, not all of these studies took into account possible confounding variables such as the effects of body mass and non-independence of different species due to phylogenetic relatedness (Page et al., 2010). To address these potential shortcomings, one study examined the rates of hydrogen peroxide generation by heart mitochondria isolated from pairs or groups of two classes of vertebrate homeotherms with similar body mass, but very different lifespan (Lambert et al., 2007). As with previous studies, the results showed a negative correlation between the rate of H_2_O_2_ generation and lifespan.

Although data from comparative studies supports the idea that lower rates of mtROS generation correlate with a greater lifespan across species, there are certain exceptions. For example, *Heterocephalus glabe* (the naked mole-rat) is the longest living rodent species known to date, with an lifespan upward of 30 years (Dammann, 2017), yet it produces similar levels of mtROS to mice, who have a lifespan of less than 4 years (Munro et al., 2019). This exception may result from the fact that naked mole-rats also have increased antioxidant defenses, which may compensate for their elevated levels of ROS (Munro et al., 2019). This highlights the importance of not considering ROS levels in a vacuum, as it is a fine balance between ROS production and ROS detoxification.

### Levels of Antioxidants Can Be Negatively Correlated With Longevity

As with levels of ROS, multiple comparative studies have examined the relationship between antioxidants and lifespan. The results from these studies are somewhat variable in that some studies show a negative correlation while others show a positive correlation, or no correlation (Table 2). To some extent the results are dependent on the specific antioxidant being measured and which tissue it is being measured in.

**TABLE 2:** Levels of antioxidants are negatively correlated with longevity in some comparative studies but not others.

Antioxidant being measured	Experimental animals	Range of lifespans	Finding	References
SOD, CAT, GPx, GSH	Mammals	3.5–30 years	Negative and positive correlations	Sohal et al., 1990
CAT, GPx, GSH	Mammals, amphibians, birds and trout	3.5–35 years	Negative correlation	Lopez-Torres et al., 1993
SOD, GRX	Mammals, amphibians, birds and trout	3.5–30 years	No correlation	Lopez-Torres et al., 1993
SOD, CAT, GPx, GRX	Amphibians, mammals and birds	4–35 years	Negative correlation	Pérez-Campo et al., 1994
SOD, CAT, GPx, GRX	Mammals and birds	3–122 years	No correlation	Page et al., 2010

*SOD, superoxide dismutase; CAT, catalase; GPx, glutathione peroxidase; GSH, glutathione; GRX, glutaredoxin*.

In a study examining antioxidant enzyme activity in six mammalian species, it was found that superoxide dismutase and catalase activity were positively correlated with lifespan, while glutathione concentration was negatively correlated with longevity (Sohal et al., 1990). A subsequent study examining antioxidant levels in liver tissue found that catalase, glutathione peroxidase and glutathione levels are negatively correlated with lifespan, while superoxide dismutase and glutathione reductase were not correlated with longevity (Lopez-Torres et al., 1993). Comparison of superoxide dismutase, catalase, glutathione peroxidases, and glutathione reductase between various amphibia, mammal and bird species in lung tissue revealed a negative correlation with lifespan (Pérez-Campo et al., 1994). In brain, heart and liver tissue of mammalian and avian species, CuZnSOD, glutathione peroxidase and glutathione reductase show no correlation with lifespan in any of the three tissues (Page et al., 2010). MnSOD and catalase were observed to be positively correlated with lifespan, however, only in brain tissue (Page et al., 2010).

Analysis of a magnitude of studies, encompassing a large variety of vertebrate species and comparing antioxidant levels in tissues from multiple organs, suggests that there is an overall negative correlation between antioxidants and lifespan. In a meta-analysis of 78 correlations between endogenous antioxidants and longevity, it was found that 72 were negative, one was positive and the rest were insignificant (Perez-Campo et al., 1998). While on the surface a negative correlation between antioxidant levels and lifespan appears to contradict the FRTA because high levels of antioxidants should result in low levels of ROS, another possible interpretation is that the levels of antioxidants are regulated in response to the levels of ROS that are present, such that high levels of antioxidants are in fact an indication of high levels of ROS generation. These two possibilities can be distinguished by measuring oxidative damage, which is determined by the balance between ROS and antioxidants.

### Oxidative Damage Is Negatively Correlated With Longevity

Oxidative damage results when the levels of ROS generated exceed a cell’s antioxidant capacity. In examining the relationship between oxidative damage and lifespan, comparative studies have generally observed a negative correlation (Table 3).

**TABLE 3:** Levels of oxidative damage are negatively correlated with longevity in comparative studies.

Type of oxidative damage	Experimental animals	Range of lifespans	Finding	References
mtDNA	Mammals and birds	3.5–46 years	Negative correlation	Barja and Herrero, 2000
DNA and Lipids	Bivalves	36–500 years	Negative correlation	Blier et al., 2017
Lipids	Mammals	3.5–46 years	Negative correlation	Pamplona et al., 2002
Lipids	Mice and birds	3.5–24 years	Negative correlation	Pamplona et al., 1999
Lipids	Rats and pigeons	4–35	Negative correlation	Pamplona et al., 1996

*mtDNA, mitochondrial DNA*.

Reactive oxygen species can oxidatively damage many biomolecules such as DNA, protein and lipids (Figure 2). The two biomolecules which show the greatest correlation with lifespan are membrane fatty acids and mtDNA (Barja, 2002). mtDNA is located in close proximity to the site of mtROS production and is therefore highly susceptible to damage during aging, leading to large deletions over time (Kowald and Kirkwood, 2018). This accumulation of oxidative damage during the aging process implicated mtDNA as a potential regulator of lifespan. In support of this idea, oxidative damage to mtDNA in the heart and brain, estimated by levels of 8-hydroxy-2-deoxyguanosine (8-oxodG), negatively correlates with lifespan in mammals and birds (Barja and Herrero, 2000). Additionally, despite extensive oxidative damage occurring to nuclear DNA (nDNA) (Richter et al., 1988), the same correlation with lifespan is not observed (Barja, 2004). Since the rate of repair of 8-oxodG is rapid in both the mitochondria and the nucleus (Anson et al., 1998), increased 8-oxodG levels in the mitochondria are due to higher rates of oxidative attack, not an inefficient repair mechanism (Barja, 2000).

Studies looking at oxidative damage to mtDNA in bivalves and other marine species have also shown a negative correlation with lifespan. The longest living non-colonial animal, *Arctica islandica*, has a lifespan of up to 400 years and provides an excellent opportunity to study aging in distant taxonomic groups. Studies using *A. islandica* find low rates of ROS generation, low levels of oxidative damage to DNA and a lower mitochondrial membrane peroxidation index compared to short-lived bivalves in the same subclass – Heteroconchia (Blier et al., 2017). Additionally, comparison between *A. islandica* and a shorter lived bivalve from the same subclass, *Mercenaria mercenaria* (lifespan of 106 years), revealed increased oxidative stress resistance associated with the species’ heightened longevity (Ungvari et al., 2011). Previous correlations between oxidative damage to mtDNA and lifespan, found in mammals and avian species, have further been corroborated in *Sebastes* (rockfish) (Hua et al., 2015).

The degree of fatty acid unsaturation in mitochondrial and cellular membranes is another parameter negatively correlated with both lifespan and oxidative stress. Unsaturated fatty acids possess extremely unstable electrons near the double bonds, making them highly susceptible to oxidative damage – a sensitivity that increases exponentially as a function of the number of double bonds per molecule (Barja, 2002). Studies have shown that the degree of fatty acid unsaturation is lower in long-lived animals than in short-lived (Pamplona et al., 2002). This adaptation in long-lived animals may be advantageous as it decreases their sensitivity to lipid peroxidation, in turn protecting other molecules from lipoxidation-derived damage (Pamplona and Barja, 2007).

A study in *Drosophila melanogaster* found that flies that developed under larval crowding had a shorter lifespan and a greater proportion of unsaturated fatty acids (Moghadam et al., 2015). They found a significant negative correlation between lifespan and the peroxidation index. Similar results have been found in studies comparing unsaturated fatty acid content and lipid peroxidation levels between mammalian and avian species (Pamplona et al., 1996, 1999; Portero-Otín et al., 2001). Despite high metabolic rates, birds, on average, have an increased lifespan compared to mammals of a similar size. Fatty acid unsaturation and lipid peroxidation are shown to be lower in parakeets and canaries compared to the mouse, protecting their tissues and organelles against free radical-induced lipid peroxidation and possibly contributing to their slower rate of aging (Pamplona et al., 1999). Similar results are obtained in studies comparing liver mitochondria of rats and pigeons (Pamplona et al., 1996) and mammals (Portero-Otín et al., 2001). The increasing evidence supporting a causal role for membrane fatty acid composition has led to an expansion of the “oxidative stress” theory of aging, described by some as the “membrane pacemaker” theory of aging (Hulbert, 2005).

Bats have been shown to live much longer than is predicted by their size and metabolic rates. While these mammals can live up to 40 years, similarly sized mice rarely live longer than 4 years. In comparing oxidative damage between bats and mice, it was found that bats have decreased protein carbonylation (Salmon et al., 2009). Bats also show decreased ROS production compared to the much shorter lived mice and shrews, though only as a ratio of oxygen consumption (Brunet-Rossinni, 2004).

Despite strong evidence demonstrating a negative correlation between oxidative damage and lifespan, there are certain exceptions. The naked mole-rat produces high levels of mtROS and accumulates significant levels of oxidative damage (Saldmann et al., 2019) and yet has a lifespan 10-fold greater than that of a similarly sized laboratory mouse (Austad, 2018). In fact, naked mole rates accrue greater oxidative damage to lipids (2-fold), mtDNA (2-fold), nDNA (8-fold) and proteins (1.5- to 2-fold) than physiologically age-matched mice (Andziak et al., 2006). Similarly, long-lived strains of *Drosophila*, vampire bats and birds have also been observed to have high levels of oxidative damage (Buffenstein et al., 2008).

## Role of ROS in Determining Longevity: Experimental Manipulation of ROS Levels

While data from comparative studies provides a useful starting point for understanding the relationship between ROS and aging, these studies are only able to demonstrate correlation, not causation. In order to determine the extent to which ROS contribute to aging, it is necessary to experimentally manipulate the levels of ROS and measure the resulting effect on lifespan. In these experimental studies the levels of ROS have been increased indirectly by knocking out genes encoding antioxidant enzymes, or directly through exposure to a ROS-generating compound. Similarly, ROS levels have been decreased by overexpressing antioxidant enzymes, or treatment with an antioxidant compound. While these types of experiments allow researchers to make conclusions about the causative role of ROS in lifespan, these studies have only been performed in a limited number of model organisms, all of which are relatively short-lived. Nonetheless, experiments involving the manipulation of ROS levels have been important in demonstrating that ROS can have beneficial or detrimental effects on longevity.

### Lifespan of Animal Models With Genetic Manipulations of Antioxidant Expression

In order to examine the effect of ROS on lifespan, researchers have generated transgenic and knockout animals that express increased or decreased levels of antioxidant enzymes and then quantified the resulting effect on lifespan. While increasing or decreasing levels of antioxidants would be predicted to decrease or increase ROS levels, it is important to measure the resulting effect on ROS levels to make firm conclusions about ROS and aging. In the case of disrupting antioxidant genes, it is possible that redundancy or a compensatory upregulation of other antioxidant genes leads to little or no change in ROS levels. In the case of overexpression of antioxidant genes, it is possible that the normal level of antioxidant gene expression is already in excess of the amount of ROS being produced such that increasing antioxidant levels has no effect.

#### Superoxide Dismutase

The effect of increasing or decreasing the levels of SOD on lifespan has been examined in yeast, worms, flies and mice. Overexpression of either cytoplasmic or mitochondrial SOD resulted in increased lifespan in yeast, worms and flies (Table 4) (Melov et al., 2000; Fabrizio et al., 2003; Harris et al., 2005; Doonan et al., 2008; Cabreiro et al., 2011). Interestingly, however, the increase in lifespan does not seem to be attributable to decreased oxidative damage, at least in worms, as worms overexpressing *sod-1* show increased levels of protein oxidation and hydrogen peroxide *in vivo* (Cabreiro et al., 2011), as well as increased sensitivity to paraquat (Doonan et al., 2008) compared to wild-type worms. In addition, the results in *Drosophila* are dependent on the strain background as overexpression of cytoplasmic SOD with catalase alone or in combination with mitochondrial SOD failed to increase lifespan in a relatively long-lived strain background (Orr et al., 2003). In contrast to the other model organisms, overexpression of cytoplasmic SOD, mitochondrial SOD or cytoplasmic and mitochondrial SOD together in mice has no effect on longevity (Huang et al., 2000; Pérez et al., 2009).

**TABLE 4:** Overexpressing antioxidant enzymes can increase or decrease lifespan.

Antioxidant Enzyme	Location	Organism	Effect on lifespan	References
Superoxide dismutase	Cytoplasm (*SOD1*)	Yeast	Increase	Harris et al., 2005
	Cytoplasm (*sod-1*)	Worms	Increase	Doonan et al., 2008
	Cytoplasm (*Sod1*)	Flies	Increase	Sun and Tower, 1999
	Cytoplasm (*Sod1*)	Mice	No effect	Huang et al., 2000
	Mitochondria (*SOD2*)	Yeast	Increase	Fabrizio et al., 2003
	Mitochondria (*sod-2*)	Worms	Increase	Melov et al., 2000
	Mitochondria (*Sod2*)	Flies	Increase	Curtis et al., 2007
	Mitochondria (*Sod2*)	Mice	No effect	Pérez et al., 2009
Catalase	Peroxisome (*CTT1*)	Yeast	Decrease	Fabrizio et al., 2003
	Peroxisome (*Cat*)	Flies	Decrease	Sun and Tower, 1999
	Cytoplasm and peroxisome (*ctl-1,ctl-2*, and *ctl-3*)	Worms	No effect	Cabreiro et al., 2011
	Peroxisome (*Cat*)	Mice	No effect	Pérez et al., 2009
	Mitochondria (*Cat*)	Mice	Increase	Schriner et al., 2005
Peroxiredoxin	Cytoplasm (*TSA1*)	Yeast	Increase	Hanzen et al., 2016
	Cytoplasm (*Prx2*)	Flies	Increase	Lee et al., 2009
	Endoplasmic reticulum (*Prx4*)	Flies	Increase	Klichko et al., 2016
	Cytoplasm, nucleus and mitochondria (*Prx5*)	Flies	Increase	Radyuk et al., 2009
Thioredoxin	Cytoplasm and nucleus (*trx-1*)	Worms	Increase	Miranda-Vizuete et al., 2006
	Nucleus (*TrxT*)	Flies	Increase	Umeda-Kameyama et al., 2007
	Cytoplasm and nucleus (*Trx-1*)	Flies	Increase	Oberacker et al., 2018
	Cytoplasm and nucleus (*Trx1*)	Mice	Increase in males	Pérez et al., 2011
Thioredoxin reductase	Mitochondria (*Trxr2*)	Flies	Increase	Pickering et al., 2017
Glutaredoxin	Exogenous	Worms	Increase	Li et al., 2018
Glutathione *S*-transferase	Cytoplasm (*gst-10*)	Worms	Increase	Ayyadevara et al., 2005
	Cytoplasm (*gst-4*)	Worms	No effect	Leiers et al., 2003
	Cytoplasm (*GstS1*)	Flies	Increase	Simonsen et al., 2008

Deletion of cytoplasmic SOD decreases lifespan in yeast, worms, flies and mice (Table 5) (Phillips et al., 1989; Elchuri et al., 2005; Ünlü and Koç, 2007; Doonan et al., 2008). Disruption of mitochondrial SOD also decreases lifespan in yeast and flies (Duttaroy et al., 2003; Ünlü and Koç, 2007). In contrast, deletion of the mitochondrial *sod* gene, *sod-2*, results in a significant increase in lifespan in *Caenorhabditis elegans* (Van Raamsdonk and Hekimi, 2009). In mice, it has not been possible to examine the effect of knocking out *Sod2* on adult lifespan as *Sod2* knockout mice die early in development (Li et al., 1995; Lebovitz et al., 1996). However, the need for *Sod2* in early development can now be circumvented using an inducible *Sod2* knockdown mouse model (Cox et al., 2018). Examination of heterozygous *Sod2+/−* mice reveals normal longevity despite increased levels of oxidative damage (Van Remmen et al., 2003).

**TABLE 5:** Deletion of genes encoding antioxidant enzymes can increase or decrease longevity.

Enzyme	Location	Organism	Effect on lifespan	References
Superoxide dismutase	Cytoplasm (*SOD1*)	Yeast	Decrease	Ünlü and Koç, 2007
	Cytoplasm (*sod-1*)	Worms	Decrease	Doonan et al., 2008
	Cytoplasm (*Sod1*)	Flies	Decrease	Phillips et al., 1989
	Cytoplasm (*Sod1*)	Mice	Decrease	Elchuri et al., 2005
	Mitochondria (*SOD2*)	Yeast	Decrease	Ünlü and Koç, 2007
	Mitochondria (*sod-2*)	Worms	Increase	Van Raamsdonk and Hekimi, 2009
	Mitochondria (*Sod2*)	Flies	Decrease	Duttaroy et al., 2003
	Mitochondria (*Sod2*)	Mice	Decrease	Li et al., 1995; Lebovitz et al., 1996
Catalase	Peroxisome (*CTA1*)	Yeast	Increase	Mesquita et al., 2010
	Peroxisome (*CTT1*)	Yeast	Increase	Mesquita et al., 2010
	Cytoplasm (*ctl-1*)	Worms	No effect	Petriv and Rachubinski, 2004
	Peroxisome (*ctl-2*)	Worms	Decrease	Petriv and Rachubinski, 2004
	Peroxisome (*catn1/catn4*)	Flies	No effect	Orr et al., 1992
	Peroxisome (*Cat*)	Mice	Decrease	Perez-Estrada et al., 2019
Glutathione peroxidase	Mitochondria (*GRX2*)	Yeast	Decrease	Managbanag et al., 2008
	Cytoplasm, nucleus and extracellular space (*gpx-1, gpx-2, gpx-6*, and *gpx-7*)	Worms	Decrease	Sakamoto et al., 2014
	Cytoplasm (*Gpx1*)	Mice	No effect	Zhang et al., 2009
	Mitochondria (*Gpx4*)	Mice	Increase	Ran et al., 2007
	Endoplasmic reticulum (*Gpx7*)	Mice	Decrease	Wei et al., 2012
Peroxiredoxin	Mitochondria (*PRX1*)	Yeast	Decrease	Molin et al., 2011
	Cytoplasm (*prdx-2*)	Worms	Decrease	Oláhová et al., 2008
	Mitochondria (*prdx-3*)	Worms	Decrease	Ha et al., 2006
	Cytoplasm, nucleus and mitochondria (*Prx5*)	Flies	Decrease	Radyuk et al., 2009
	Cytoplasm (*Prdx1*)	Mice	Decrease	Neumann et al., 2003
Thioredoxin	Cytoplasm (*TRX1*)	Yeast	Decrease	Laschober et al., 2010
	Cytoplasm (*trx-1*)	Worms	Decrease	Jee et al., 2005
	Mitochondria (*trx-2*)	Worms	No effect	Cacho-Valadez et al., 2012
	Mitochondria (*Trx2*)	Flies	Decrease	Svensson and Larsson, 2007
	Mitochondria (*Trx2*)	Mice	Decrease	Pérez et al., 2008
Thioredoxin reductase	Cytoplasm (*TRXR1*)	Yeast	Decrease	Picazo et al., 2018
	Cytoplasm (*trxr-1*)	Worms	No effect	Li et al., 2012
	Mitochondria (*trxr-2*)	Worms	No effect	Li et al., 2012
	Cytoplasm (*Trxr1*)	Flies	Decrease	Missirlis et al., 2001
Glutaredoxin	Cytoplasm (*GRX1*)	Yeast	Decrease	Liu et al., 2018
	Cytoplasm and mitochondria (*GRX2*)	Yeast	Decrease	Liu et al., 2018
	Mitochondria (*GRX5*)	Yeast	Decrease	Zadrag et al., 2008
Glutathione *S*-transferase	Cytoplasm (*gst-5*)	Worms	Decrease	Ayyadevara et al., 2007
	Cytoplasm (*gst-10*)	Worms	Decrease	Ayyadevara et al., 2007
	Microsome (*MGst1*)	Flies	Decrease	Toba and Aigaki, 2000
	Cytoplasm (*GstA4*)	Mice	Increase	Singh et al., 2010

#### Catalase

In contrast to what was observed with SOD, overexpression of catalase resulted in decreased lifespan in both yeast and flies (Table 4) (Sun and Tower, 1999; Fabrizio et al., 2003), despite the fact that catalase overexpression increased resistance to oxidative stress (Sun and Tower, 1999). In worms, overexpression of all three catalase genes combined (*ctl-1, ctl-2*, and *ctl-3*) did not affect lifespan but resulted in a paradoxical increase in oxidative damage to proteins (Cabreiro et al., 2011). In mice, overexpression of catalase in the peroxisome, where catalase is normally found, did not affect longevity (Pérez et al., 2009), while overexpression of catalase targeted to the mitochondria did increase lifespan (Schriner et al., 2005).

Simultaneous overexpression of cytosolic SOD and catalase was initially reported to extend lifespan in *Drosophila* (Orr and Sohal, 1994), but when these experiments were replicated, there was no significant change in lifespan (Sun and Tower, 1999). Similar to the latter experiment, simultaneous overexpression of cytoplasmic SOD and catalase in mice did not affect lifespan (Pérez et al., 2009). Simultaneous overexpression of mitochondrial SOD and catalase resulted in decreased lifespan in *Drosophila* (Bayne et al., 2005).

Inactivation of catalase in yeast resulted in increased lifespan, despite also increasing oxidative damage (Table 5) (Mesquita et al., 2010). In *C. elegans*, deletion of cytoplasmic catalase has no effect on lifespan, while loss of peroxisomal catalase decreases lifespan (Petriv and Rachubinski, 2004). In *Drosophila*, complete loss of catalase activity does not affect longevity (Orr et al., 1992). Finally, disruption of catalase in mice results in decreased lifespan (Perez-Estrada et al., 2019).

#### Glutathione Peroxidase

There have been only a limited number of studies examining the effects of glutathione peroxidase modulation on lifespan. Deletion of *gpx2* in yeast results in decreased lifespan (Managbanag et al., 2008). In *C. elegans*, simultaneous deletion of four intestinal *gpx* genes decreases lifespan, despite having no effect on resistance to oxidative stress (Table 5) (Sakamoto et al., 2014). In mice, deletion of *Gpx1*, one of the most abundant glutathione peroxidases, increases oxidative damage but does not significantly affect longevity, even when combined with a heterozygous mutation in *Sod2* (Zhang et al., 2009). Interestingly, heterozygous mice that have a 50% reduction in *Gpx4* levels have increased lifespan compared to wild-type mice (Ran et al., 2007). Finally, it has been shown that disruption of *Gpx7* in mice results in decreased lifespan, which is associated with increased oxidative stress (Wei et al., 2012). Interestingly, GPX7 lacks enzymatic activity and instead acts as a stress sensor (Chen et al., 2016).

#### Peroxiredoxin

Single copy overexpression of the yeast cytosolic peroxiredoxin gene, *tsa1*, is sufficient to increase lifespan (Table 4) (Hanzen et al., 2016). Overexpression of either *Prx2*(*Jafrac1*) or *Prx5* in flies also increases resistance to oxidative stress and lifespan (Lee et al., 2009; Radyuk et al., 2009). A moderate overexpression of *Prx4* reduces oxidative damage and extends lifespan in flies, but high levels of expression shortens longevity (Klichko et al., 2016).

Deletion of the yeast cytosolic peroxiredoxin, *tsa1*, decreases lifespan (Table 5) (Molin et al., 2011). While loss of *Tsa1* also increases sensitivity to hydrogen peroxide, this does not contribute to the shortening of lifespan, which instead is attributed to the role of *Tsa1* as an inhibitor of nutrient signaling via protein kinase A (Roger et al., 2020). Others have reported that disruption of all five yeast peroxiredoxin genes does not decrease lifespan (Zadrag et al., 2008). In *C. elegans*, deletion of the cytoplasmic peroxiredoxin, *prdx-2*, results in increased sensitivity to hydrogen peroxide and decreased lifespan (Oláhová et al., 2008). Interestingly, expression of PRDX-2 in the intestine of *prdx-2* deletion mutants eliminates sensitivity to hydrogen peroxide but fails to restore lifespan (Oláhová et al., 2008) suggesting that the decrease in lifespan may not be attributable to the increase in sensitivity to oxidative stress. As with *prdx-2*, knockdown of mitochondrial peroxiredoxin, *prdx-3*, in *C. elegans* using RNAi resulted in decreased lifespan (Ha et al., 2006). In *Drosophila*, deletion of *Prx5* (Radyuk et al., 2009) or *Prx3* and *Prx5* together (Orr et al., 2013) increases susceptibility to oxidative stress and decreases lifespan. Finally, deletion of *Prx1* in mice increases ROS production and oxidative damage and decreases lifespan (Neumann et al., 2003; Rani et al., 2012).

#### Thioredoxin and Thioredoxin Reductase

In *C. elegans*, overexpression of the cytoplasmic thioredoxin gene *trx-1* moderately increases lifespan (Table 4) (Miranda-Vizuete et al., 2006). Similarly, overexpression of *TrxT* in flies is also found to increase lifespan (Umeda-Kameyama et al., 2007). Downregulation of thioredoxin-interacting protein (TXNIP), a negative regulator or *Trx1*, increases resistance to oxidative stress and extends lifespan, while overexpression decreases lifespan (Oberacker et al., 2018). While one group reported that overexpressing mitochondrial thioredoxin reductase significantly increases lifespan in flies (Pickering et al., 2017), it has also been reported that overexpression of thioredoxin reductase in combination with CuZnSOD and catalase does not affect longevity (Orr et al., 2003). While an earlier study found that overexpression of human thioredoxin in mice resulted in decreased oxidative stress and increased lifespan (Mitsui et al., 2002), subsequent studies observed less beneficial or even detrimental effects. Overexpression of *Trx1* in mice was found to only have significant effects on lifespan in male, but not female, mice, and only increased the earlier part of lifespan (Pérez et al., 2011; Flores et al., 2018). Interestingly, simultaneous overexpression of *Trx1* and *Trx2* resulted in significantly decreased lifespan (Cunningham et al., 2018).

Deletion of the gene encoding thioredoxin in yeast, *trx1*, slightly reduces chronological lifespan (Table 5) (Laschober et al., 2010). Deletion of the yeast cytosolic thioredoxin reductase, *trr1*, also reduces chronological lifespan and increases sensitivity to oxidative stress (Picazo et al., 2018). In *C. elegans*, disruption of the cytoplasmic thioredoxin gene, *trx-1*, causes a significant decrease in lifespan (Jee et al., 2005), while deletion of the mitochondrial thioredoxin gene, *trx-2* has no effect (Cacho-Valadez et al., 2012). In contrast, disruption of the cytoplasmic (*trxr-1*) or mitochondrial (*trxr-2*) thioredoxin reductase genes, or both together, did not affect lifespan at the normal growing temperature of 20°C (Li et al., 2012). In flies, mutations affecting *Trx2* shortens lifespan and increases sensitivity to oxidative stress (Svensson and Larsson, 2007). Unlike *C. elegans*, mutations that reduce thioredoxin reductase activity in *Drosophila* cause a severe reduction in adult lifespan (Missirlis et al., 2001). In mice, a 50% reduction in expression of the mitochondrial thioredoxin gene, *Trx2*, increases ROS production and oxidative damage and slightly reduces lifespan (Pérez et al., 2008).

#### Glutaredoxin

Relatively few studies have examined the effect of glutaredoxins on longevity. Intake of recombinant buckwheat glutaredoxin in *C. elegans* increases lifespan and resistance to oxidative stress (Table 4) (Li et al., 2018). Deletion of either *grx1* or *grx2* in yeast shortens the chronological lifespan via increased ROS accumulation which subsequently activates the RAS/PKA signaling pathway and decreases stress resistance (Table 5) (Liu et al., 2018). Disruption of the mitochondrial glutaredoxin gene, *grx5*, also decreases lifespan in yeast (Zadrag et al., 2008).

#### Glutathione *S*-Transferase

Despite the fact that there are many different glutathione *S*-transferases (e.g., 57 in *C. elegans*), very few have been shown to influence lifespan, possibly due to functional redundancy with other glutathione *S*-transferases. In *C. elegans*, overexpression of *gst-4* increases resistance to oxidative stress but does not increase lifespan (Leiers et al., 2003), while overexpression of *gst-10* decreased oxidative damage (4-HNE) and increased lifespan (Table 4) (Ayyadevara et al., 2005). In *Drosophila*, overexpression of *GstS1* significantly increases mean lifespan and resistance to oxidative stress (Simonsen et al., 2008).

Decreasing the expression of either *gst-5* or *gst-10* causes decreased lifespan in *C. elegans* (Table 5) (Ayyadevara et al., 2007). In contrast, deletion of *gst-14* has the opposite effect of increasing lifespan, although its effects were only reported in mutants that have impaired mitochondrial function (Suthammarak et al., 2013). In *Drosophila*, deletion of microsomal glutathione *S*-transferase was found to reduce lifespan (Toba and Aigaki, 2000). In mice, deletion of *GstA4*, a glutathione *S*-transferase important for detoxification of the lipid peroxidation product 4-hydroxynonenal (4-HNE), extends mean lifespan (Singh et al., 2010).

In summary, genetic manipulations of antioxidant gene expression have varied effects on the lifespan of model organisms. While the general trend is that overexpression of antioxidant enzymes increases lifespan and disruption of antioxidant genes decreases lifespan, there are multiple examples in which no effect or the opposite effect is observed. The ability of antioxidant gene manipulation to significantly change lifespan is dependent on a number of factors including the species in which the manipulation is taking place, the specific antioxidant being altered, and the subcellular location in which the antioxidant is expressed.

### Effect of Antioxidant Compounds on Lifespan

Treatment with antioxidant compounds is another approach to studying the relationship between ROS and lifespan. While this approach is an indirect way of modulating ROS levels, it can be applied to a wide range of species, including humans. Nonetheless, experiments using antioxidant compounds have generally been performed in short-lived species so that experiments can be completed in a feasible time frame. Because this approach is indirect, it is important to measure the effect of the antioxidant compound on the levels of ROS in order to fully interpret the results. In addition, when performing experiments with compounds, it is important to consider that most or all of these antioxidant compounds have other effects aside from their antioxidant activity that may contribute to or account for their effects on lifespan. Experiments using this approach have provided mixed results as to the effect of antioxidant supplementation on lifespan (Table 6).

**TABLE 6:** Exposing animals to antioxidant compounds can increase or decrease lifespan.

Compound	Organism	Dose	Effect on lifespan	References
*N*-acetyl cysteine	Worms	9 mM	Increase	Oh et al., 2015
	Flies	1 mg/ml	Increase	Brack et al., 1997
	Mice	10 g/L	Increase in males	Flurkey et al., 2010
Vitamin C	Yeast	20 mM	Increase	Owsiak et al., 2010
	Worms	0.24 mg/liposome	Increase	Shibamura et al., 2009
	Flies	20 mM	Increase	Bahadorani et al., 2008
	Mice	57 mM	Increase	Massie et al., 1984
	Wild-derived voles	180 mg/kg	Decrease	Selman et al., 2013
	Guinea pigs	57 mM	No change	Davies et al., 1977
Vitamin E	Yeast	0.05 mM	Decrease	Lam et al., 2010
	Worms	0.2 mg/ml	Increase	Harrington and Harley, 1988
	Flies	20 μg/ml	Increase	Driver and Georgeou, 2003
	Mice	20–4,000 μg/g	No change	Morley and Trainor, 2001
	Wild-derived voles	550 mg/kg	Decrease	Selman et al., 2013

#### *N*-Acetyl Cysteine

*N*-acetyl cysteine (NAC) is a scavenger of free radicals as well as a precursor of L-cysteine and a source of sulfhydryl groups (Karalija et al., 2012). It derives most of its antioxidant properties as a cysteine precursor, promoting the synthesis of glutathione. Treatment of *C. elegans* with NAC shows a U-shaped dose-response curve in which low concentrations of NAC can increase lifespan, while higher concentrations decrease lifespan (Oh et al., 2015; Desjardins et al., 2017). A similar pattern is observed in *Drosophila* where lower concentrations of NAC can increase lifespan, while higher concentrations shorten lifespan (Brack et al., 1997; Niraula and Kim, 2019). The effect of NAC treatment in mice is sex dependent as NAC was found to only increase lifespan in males, but not in females (Flurkey et al., 2010). It is important to note, however, that mice treated with NAC show decreases in body weight making it possible that the increase in lifespan is due to dietary restriction (Flurkey et al., 2010).

#### Vitamin C

Vitamin C is a hydrophilic antioxidant and a strong inhibitor of lipid peroxidation (Sadowska-Bartosz and Bartosz, 2014). Vitamin C treatment in yeast was found to increase survival in wild-type strains (Owsiak et al., 2010). Treatment of *C. elegans* with Vitamin C has also been shown to increase wild-type lifespan when delivered with liposomes (Shibamura et al., 2009) or when the *C. elegans* cuticle is made more permeable with a *bus-8* mutation (Desjardins et al., 2017). Vitamin C supplementation in *Drosophila* can extend the lifespan of wild-type flies, while high doses of Vitamin C can be toxic and shorten lifespan (Bahadorani et al., 2008). Vitamin C treatment in rodents has provided somewhat mixed results. Treating lab mice with Vitamin C resulted in a slight increase in lifespan (Massie et al., 1984). In contrast, Vitamin C treatment shortened the lifespan of wild-derived voles (Selman et al., 2013). Additionally, Vitamin C treatment in guinea-pigs had no effect on lifespan (Davies et al., 1977).

#### Vitamin E

Vitamin E is a lipophilic antioxidant that protects membranes from oxidative damage as well as regulating signal transduction and gene expression. Vitamin E supplementation in yeast is observed to shorten replicative lifespan (Lam et al., 2010) despite increasing the mean lifespan of four other single cell organisms (Ernst et al., 2013). Multiple groups have shown that Vitamin E treatment can increase lifespan in *C. elegans* (Zuckerman and Geist, 1983; Harrington and Harley, 1988), but interestingly this treatment was found to have no effect on superoxide levels (Ishii et al., 2004). While some groups have reported that Vitamin E treatment increases lifespan in *Drosophila*, others have observed little or no effect on longevity (Driver and Georgeou, 2003; Zou et al., 2007; Bahadorani et al., 2008). Vitamin E treatment in mice is largely observed to have no significant effect on lifespan (Lipman et al., 1998; Morley and Trainor, 2001) but was found to significantly increase lifespan in male mice in one study (Navarro et al., 2005). In contrast, Vitamin E supplementation was found to decrease lifespan in wild-derived voles (Selman et al., 2013).

#### Vitamin Supplementation Does Not Decrease Mortality in Humans

A number of randomized clinical trials have examined the effects of different antioxidants in humans, including Vitamin C, Vitamin E, Vitamin A, beta-carotene and selenium. A meta-analysis of 78 trials that included a total of 296,707 individuals showed no beneficial effect of any of the antioxidants for human longevity in control or disease populations (Bjelakovic et al., 2012, 2013). In fact, treatment with Vitamin E or beta carotene resulted in a higher risk of all-cause mortality. While the average length of supplementation was 3 years, these results suggest that decreasing ROS levels may not be beneficial for lifespan in people.

## A Mild Increase in Levels of Reactive Oxygen Species Can Extend Longevity

### Exposure to a Mild Dose of ROS-Generating Compounds Increases Lifespan

Another approach to examine the relationship between ROS and aging is to expose animals to compounds that act to generate ROS. Although this approach can be applied to any species, these experiments have only been performed in relatively short-lived species for practical reasons. While exposing animals to high levels of ROS-generating compounds is toxic, evidence from multiple species indicates that mildly increasing ROS levels with ROS-generating compounds can extend longevity (Table 7).

**TABLE 7:** Examples of ROS-generating compounds that have been shown to increase lifespan.

Compound	Organism	Dose	Effect on lifespan	References
2-deoxy-D-glucose	Worms	5 mM	Increase	Schulz et al., 2007
Juglone	Worms	40 μM	Increase	Heidler et al., 2010
Paraquat	Worms	0.1 mM	Increase	Yang and Hekimi, 2010
Plumbagin	Worms	25 μM	Increase	Hunt et al., 2011
Menadione	Yeast	1 μM	Increase	Pan et al., 2011
Rotenone	Worms	100 nM	Increase	Schmeisser et al., 2013a
Arsenite	Worms	0.1 μM	Increase	Schmeisser et al., 2013b
Metformin	Worms	50 mM	Increase	De Haes et al., 2014
D-glucosamine	Worms	100 μM	Increase	Weimer et al., 2014
	Mice	10 g/kg	Increase	Weimer et al., 2014

One of the first experiments to show that compounds that increase ROS levels can extend longevity involved treating *C. elegans* with 2-deoxy-D-glucose (Schulz et al., 2007). 2-deoxy-D-glucose acts as an inhibitor of glycolysis, leading to increased mitochondrial respiration, increased production of ROS and increased lifespan. Importantly, the increase in ROS levels was found to be required for the lifespan extension as the increase in longevity was prevented by treatment with antioxidants (NAC, Vitamin C or Vitamin E). Similarly, treatment of *C. elegans* with arsenite results in increased ROS and extended longevity, which is completely dependent on the increase in ROS, as treatment with antioxidants (NAC, BHA[butylated hydroxyanisole]) reverts lifespan to control (Schmeisser et al., 2013a). In *C. elegans*, inhibition of mitochondrial electron transport chain Complex I with rotenone causes an increase in mitochondrial ROS and extends lifespan (Schmeisser et al., 2013a). Metformin has also been shown to increase worm lifespan through a similar mechanism. It was found that metformin treatment led to an inhibition of Complex I, increased respiration, increased ROS and extended longevity, which was prevented by treatment with the antioxidant NAC (De Haes et al., 2014). It has also been shown that treating worms with either juglone or paraquat, which act to directly increase superoxide levels primarily in the mitochondria, extends longevity (Heidler et al., 2010; Yang and Hekimi, 2010).

The ability of ROS-generating compounds to extend lifespan is not limited to *C. elegans*, as treating yeast with menadione, which acts to generate mitochondrial ROS through redox cycling, was also shown to increase lifespan (Pan et al., 2011). Interestingly, menadione did not increase lifespan in *C. elegans* (Hunt et al., 2011; Urban et al., 2017). Similarly, treatment with paraquat or metformin failed to extend lifespan in *Drosophila* (Slack et al., 2012; Scialo et al., 2016). The reason why these ROS-generating compounds increase lifespan in some species but not others is currently unclear. Since these compounds have the potential to be toxic at higher doses, it is possible that the doses used were simply too high, or that other experimental parameters were not optimal for the compound to extend lifespan in the species showing no effect. It is also possible that there are differences between species in the ways that ROS can extend longevity. For example, it was proposed that compounds that affect Complex I, such as paraquat and metformin, don’t increase lifespan in *Drosophila* because an intact Complex I may be necessary for lifespan extension in this organism (Scialo et al., 2016).

Importantly, ROS-generating compounds have also been shown to increase lifespan in mammals. Similar to 2-deoxy-D-glucose, D-glucosamine acts as an inhibitor of glycolysis leading to increased respiration and increased production of ROS. Treatment of worms or mice with D-glucosamine increases lifespan, and this increase in lifespan was shown to be dependent on elevated ROS as it is prevented by treatment with antioxidants (NAC, BHA) (Weimer et al., 2014).

### Genetic Mutations That Extend Longevity by Increasing Levels of Reactive Oxygen Species

Genetic mutations that lead to increased levels of ROS have been shown to have variable effects on lifespan: they can decrease lifespan, increase lifespan or have no effect (Van Raamsdonk and Hekimi, 2010; Van Raamsdonk et al., 2010). In this section, we will focus on genetic mutants in which increased ROS causes extended longevity, providing examples from yeast, worms, flies and mice (Table 8).

**TABLE 8:** Examples in which increased levels of ROS cause increased lifespan.

Organism	Intervention	Effect	Effect on lifespan	References
Yeast	Catalase deletion	Increase ROS	Increase	Mesquita et al., 2010
Worms	*sod-2* deletion	Decrease mitochondrial superoxide detoxification	Increase	Van Raamsdonk and Hekimi, 2009
	*clk-1* mutation	Increase ROS	Increase	Schaar et al., 2015
	*nuo-6* mutation	Increase mtROS	Increase	Yang and Hekimi, 2010
	*isp-1* mutation	Increase mtROS	Increase	Yang and Hekimi, 2010
	*daf-2* mutation	Increase ROS	Increase	Zarse et al., 2012
	*glp-1* mutation	Increase ROS	Increase	Wei and Kenyon, 2016
	*memo-1* mutation	Increase ROS	Increase	Ewald et al., 2017
	Knockdown of *ero-1*	Increase ROS	Increase	Hourihan et al., 2016
Flies	Expression of NDI1 (alternative component of ETC)	Increase mtROS	Increase	Scialo et al., 2016
	Muscle-specific knockdown of ND75 component on ETC complex I	Increase ROS	Increase	Owusu-Ansah et al., 2013
Mice	Mclk1 heterozygosity	Increase mtROS	Increase	Liu et al., 2005
	Decreasing body temperature	Increase mtROS	Increase	Ristow and Schmeisser, 2011

In yeast, it has been shown that disruption of genes encoding catalase (*cta1* or *ctt1*) increases the levels of intracellular ROS (hydrogen peroxide) and increases oxidative damage (protein carbonylation), but also increases lifespan (Mesquita et al., 2010). This shows that increasing ROS can increase lifespan, and that oxidative damage can be experimentally dissociated from lifespan.

In *C. elegans*, it has been shown that deletion of the mitochondrial *sod* gene, *sod-2*, increases lifespan, despite also resulting in increased oxidative damage (Van Raamsdonk and Hekimi, 2009). The increase in lifespan in *sod-2* mutant worms is dependent on elevated ROS, as treatment with antioxidants (Vitamin C, α-lipoic acid, or epigallocatechin gallate) reverts their lifespan toward wild-type. Three different mutations that affect mitochondrial function in *C. elegans, clk-1, nuo-6*, and *isp-1*, have all been shown to extend longevity despite increasing levels of ROS (Yang and Hekimi, 2010; Schaar et al., 2015; Dues et al., 2017). As with *sod-2* mutants, treatment of these strains with antioxidants decreases their lifespan, again demonstrating that the elevation in ROS levels in these strains is required for their longevity (Yang and Hekimi, 2010; Van Raamsdonk and Hekimi, 2012). Decreasing insulin-IGF1 signaling has been shown to increase lifespan in multiple species and to be associated with longevity in humans. In *C. elegans*, disruption of this pathway with a mutation in the insulin-IGF1 receptor gene, *daf-2*, results in increased levels of ROS, which are required for their long lifespan (Zarse et al., 2012). Similarly, inhibition of the germline has been shown to increase lifespan in *C. elegans* in a ROS-dependent manner: mutations in *glp-1* prevent germline development, increase ROS levels and extend longevity (Wei and Kenyon, 2016).

In *Drosophila*, expression of an alternative component of the mitochondrial electron transport chain called NDI1 causes increased production of ROS during mitochondrial electron transport. This increase in ROS is sufficient to extend the longevity of the fly, and is prevented by overexpression of catalase (Scialo et al., 2016). Also in *Drosophila*, it has been shown that muscle-specific knockdown of a subunit of electron transport chain Complex I called ND75 results in increased levels of ROS and increased lifespan (Owusu-Ansah et al., 2013). Overexpression of either catalase or glutathione peroxidase reverted the lifespan of ND75-knockdown flies to wild-type, thereby indicating that the increase in ROS is required for their long lifespan (Owusu-Ansah et al., 2013).

In mice, it has been shown that a heterozygous mutation in the mouse homolog of *clk-1, Mclk1*, results in increased levels of mitochondrial ROS, increased levels of oxidative damage and increased lifespan (Liu et al., 2005). Decreasing body temperature has been shown to increase ROS and to extend longevity in mice (Conti et al., 2006; Ristow and Schmeisser, 2011).

Combined, the experiments described above demonstrate that genetic mutations that lead to increased levels of ROS can extend longevity in yeast, worms, flies and mice. Importantly, in most of these experiments the lifespan-extending effect of the mutation was prevented by treatment with antioxidants, thereby indicating that the elevated levels of ROS are required for the observed lifespan extension.

### ROS-Mediated Pro-survival Signaling

While the reactive nature of ROS can be perceived as making them dangerous to cells, it is also possible for cells to harness this property to use ROS as an efficient tool for intracellular signaling (de Magalhaes and Church, 2006). ROS have been shown to act as signaling molecules for a number of biological processes (Sena and Chandel, 2012; Reczek and Chandel, 2015; Sies and Jones, 2020), including development (Covarrubias et al., 2008) and survival (Groeger et al., 2009). ROS can oxidize cysteine residues within proteins leading to allosteric changes that alter that protein’s function. Those cysteine residues can subsequently be reduced by thioredoxin or glutaredoxin to restore their original function or be further oxidized in a manner that is irreversible. In this way, ROS can activate kinase signaling pathways through inactivation of phosphatases or turn off kinase signaling pathways by inactivating kinases. Similarly, ROS can act directly on effector proteins to mediate their effects, such as inhibiting cell death proteins to promote survival. ROS involved in intracellular signaling can be generated by enzymes such as NADPH oxidases (Lambeth, 2004). In fact, there are over 40 enzymes that generate hydrogen peroxide or superoxide in human cells (Sies and Jones, 2020). In the context of signaling, the ROS generated from the electron transport chain may be a cell’s way of communicating the status of the electron transport chain function to the rest of the cell.

The ability for redox-signaling proteins to switch between oxidized and reduced states is essential for many of the signaling pathways mentioned. The redox environment in the cell, as determined by levels of ROS and antioxidants, determines the balance between proteins in reduced and oxidized states, also known as the redox poise of a protein. Maintaining an optimal state of redox poise is important not only for signaling molecules that respond to changes within the redox environment, but also for optimal transfer of electrons in the electron transport chain. This notion that both over-reduction and over-oxidation of cell components can be detrimental to cell function supports the hypothesis that optimal levels of ROS and not simply low levels of ROS are required to maintain homeostasis (Allen, 2004).

The fact that ROS are involved in intracellular signaling and cause oxidative damage highlights the crucial importance of maintaining ROS at an optimal level, and provides a mechanism for how ROS act to extend longevity. There appears to be a “goldilocks” zone in which the beneficial effects of ROS-mediated signaling are maximized while minimizing the amount of oxidative damage caused by ROS (Alleman et al., 2014).

## Discussion

The FRTA provides a conceptual framework that is used to think about the relationship between ROS and aging, as well as specific predictions that can be experimentally tested. At its most basic level, the FRTA posits that aging is driven by the accumulation of ROS-induced macromolecular damage. If the FRTA is correct then (1) levels of oxidative damage should be negatively correlated with lifespan, (2) experimental manipulations that decrease oxidative damage should increase longevity, and (3) experimental manipulations that increase oxidative damage should shorten lifespan.

Comparative studies generally suggest that the levels of ROS and oxidative damage are negatively correlated with lifespan (Table 1 and Table 3). While examples that do not follow this trend exist, such as the naked mole-rat, it is unclear if these examples are exceptions to the rule, or evidence that the rule is incorrect. The great strength of comparative studies is that they study a diverse set of species with lifespans ranging from days to hundreds of years. However, care must be taken when interpreting comparative data, as correlation cannot demonstrate causation. Thus, even though oxidative damage increases with age, this does not necessarily mean that it causes aging. It is also possible that different factors cause oxidative damage and aging but that both increase over time, or that the same factor causes both, but does so independently. In order to distinguish between these possibilities, it is necessary to experimentally modulate the proposed causative factor and measure the resulting effect on lifespan.

A number of approaches have been utilized to experimentally modulate the levels of ROS or oxidative damage to determine the effect of the intervention on longevity. In general, interventions aimed at decreasing ROS levels, including overexpression of antioxidant enzymes or treatment with antioxidant compounds, have been shown to increase lifespan, though there are also examples where no effect or a decrease in lifespan is observed (Table 4). While theoretically these interventions should decrease oxidative damage, this is not always the case. In some cases, interventions aimed at decreasing ROS resulted in increased oxidative damage and still increased lifespan, thereby indicating that the underlying mechanism of lifespan extension with interventions that decrease ROS is not necessarily a reduction in oxidative damage. In general, interventions aimed at increasing ROS levels, primarily disruption of genes encoding antioxidant enzymes, have been shown to decrease lifespan (Table 5). Nonetheless, there are also examples in which deletion of genes encoding antioxidant enzymes has no effect or results in increased lifespan, despite increasing levels of oxidative damage.

Despite the fact that the bulk of evidence suggests that ROS are detrimental for longevity, a number of experimental studies have demonstrated that ROS can also increase lifespan. This has been demonstrated in yeast, worms, flies and mice and has been shown through both genetic manipulations and compounds that increase ROS levels (Tables 7 and 8). These experiments highlight the fact that ROS can be beneficial or detrimental with respect to longevity. The effect of ROS on lifespan is determined by a number of factors including which type of ROS it is (e.g., superoxide and hydrogen peroxide), which subcellular compartment it is in (e.g., mitochondria and cytoplasm), which tissue it is in (e.g., neurons and muscle), when during the life cycle it is increased (e.g., development and adulthood) and how much the ROS levels are increased. The mechanisms by which ROS increase lifespan are likely mediated by their ability to participate in ROS-mediated signaling to activate genetic pathways that increase survival (e.g., Senchuk et al., 2018; Wu et al., 2018).

There are two important caveats for experimental studies. First, genetic studies involving overexpression or deletion of antioxidant genes have only been performed in four well-characterized genetic model organisms: yeast, worms, flies and mice. It is unclear to what extent findings in these four species are reflective of the wide diversity of organisms across the tree of life. The advance of genome sequencing technologies and genetic engineering tools such as CRISPR-Cas9 will make it easier to manipulate gene expression in other organisms, thereby enabling researchers to determine if the results from these four organisms are representative of a wider range of species. Given that different outcomes have already been observed between these four species, it is uncertain if general patterns will emerge when studying a broader range of species.

The second major caveat of experimental studies, both genetic and pharmacological, is the fact that these studies have primarily been performed in short-lived species. In order to test the effect of a gene or compound on the lifespan of an organism, practicalities make it challenging to perform experiments in species that live longer than 5 years, which is the length of a typical grant and roughly the duration of a doctoral program. Thus, one cannot exclude the possibility that ROS affect lifespan differently in short-lived and long-lived species.

Perhaps one of the greatest limitations of both comparative and experimental studies examining the effect of ROS on lifespan is the need to measure ROS. Comparative studies require accurate measurement of ROS and oxidative damage to correlate with longevity. Experimental studies require precise measurement of ROS levels to determine whether the experimental manipulation is having the desired effect. However, the instability and reactivity of ROS make them extremely difficult to quantify. If they are not measured *in vivo*, in real-time, it is unclear whether the ROS levels being measured are truly reflective of the ROS levels in the organisms being tested, since ROS can react rapidly with cellular components, or be detoxified by antioxidants. Unfortunately, current methods to measure ROS are still limited by many confounding variables, such as limited specificity and sensitivity (Starkov, 2010; Deshwal et al., 2018). As experimental studies have demonstrated that the effect of ROS on lifespan is dependent on many factors, current approaches to measure ROS may be too blunt to distinguish between lifespan-extending ROS and lifespan-shortening ROS. This may be especially true with respect to the spatial localization of ROS within a cell. For example, elevated superoxide in the mitochondria can extend longevity through mitochondria-nucleus signaling, while increasing superoxide in other parts of the cell can shorten lifespan (Schaar et al., 2015).

Many of the studies reported here provide a limited view of the redox balance occurring in the organism being studied. Oxidative stress is a balance between ROS production and ROS detoxification by antioxidants, and this balance may change over time. However, most studies only measure one type of ROS, at one time point, in one tissue. A challenge for the field moving forward is to be able to measure and take into account all of these different factors that can influence how ROS affects longevity in a way that is experimentally feasible.

As a final note, it is important to consider the possible effects of observation bias. In cases where an experiment shows no effect on lifespan, it is unclear whether this finding is because the intervention does not affect lifespan, or because the experiment failed to demonstrate an effect of the intervention on lifespan. As a result, experiments in which the outcome shows that the intervention tested does not affect lifespan are difficult to interpret and are more likely not to be reported than experiments showing a positive or negative effect on lifespan.

## Conclusion

Despite extensive research, the role of ROS in aging remains incompletely understood. While comparative studies have demonstrated a negative relationship between both ROS levels and oxidative damage, and lifespan, these correlations do not necessarily imply causation, and exceptions to these relationships have been observed. In experimental studies, it has generally been observed that manipulations that increase ROS decrease lifespan, while manipulations that decrease ROS increase lifespan. However, there are several examples in which a manipulation’s effect on ROS levels or oxidative damage can be experimentally dissociated from its effect on lifespan. In addition, there are examples in which increasing ROS levels extends longevity, and in which decreasing ROS levels shortens lifespan. One of the greatest limitations to our understanding of the role of ROS in aging is our ability to measure ROS. There are many different types of ROS, and the exact tissue, subcellular location, timing and levels of ROS act to determine whether ROS increase or decrease lifespan. With the currently available tools to measure ROS and the present limitations of comparative and experimental studies, it may not be possible to generalize the relationship between ROS and aging across the tree of life.

## Author Contributions

JV: conceptualization and supervision. HS and AT: investigation and writing – original draft. HS, AT, and JV: visualization and writing – review and editing. All authors contributed to the article and approved the submitted version.

## Conflict of Interest

The authors declare that the research was conducted in the absence of any commercial or financial relationships that could be construed as a potential conflict of interest. The handling editor declared a past co-authorship with one of the authors JV.
